# Thyroglossal Duct Cyst Carcinoma in a Young Female: Case Report and Review of Literature

**DOI:** 10.1155/2019/4069375

**Published:** 2019-07-31

**Authors:** Jenna Van Beck, Sobia F. Khaja

**Affiliations:** Department of Otolaryngology-Head and Neck Surgery, University of Minnesota, Minneapolis, MN, USA

## Abstract

Thyroglossal duct remnants form during embryologic development and can develop into a thyroglossal duct cyst (TGDC). In rare cases, carcinoma is present within these cysts, most commonly papillary thyroid carcinoma. Diagnosis is difficult, but imaging and fine-needle aspiration (FNA) biopsies can help with the diagnosis. Given the rarity of TGDC carcinoma, treatment is not well agreed upon and can include the Sistrunk procedure, thyroidectomy, nodal dissection, and postoperative radioactive iodine treatment. Here, we describe the presentation, workup, and treatment of a 20-year-old female with papillary thyroid carcinoma present within both a thyroglossal duct cyst and the thyroid gland.

## 1. Introduction

The thyroglossal duct develops during thyroid gland formation. During development, the thyroid gland descends from the foramen cecum at the base of the tongue and migrates down to its final position in the anterior neck. The thyroglossal duct is the portion of the tract that remains connected to the base of the tongue after thyroid gland descent and is thought to involute at 7–10 weeks gestation. Persistence of portions of the thyroglossal duct can lead to formation of thyroglossal duct cysts (TGDCs), remnants, or tracts [1, 2]. The prevalence of TGDCs is thought to be roughly 7% of the population, with 1% of those developing into TGDC carcinoma. The most common type of malignancy within a thyroglossal duct cyst is papillary thyroid cancer, seen in >90% of cases [3].

TGDC carcinomas can be difficult to diagnose, as it can often look very similar or even identical to benign TGDCs. Fine-needle aspiration (FNA) has low diagnostic accuracy, and therefore, its role is debated. Rapid increase in size of the mass or a mass that is firm and palpable can be indicative of malignancy [3–5]. Imaging can sometimes indicate the possibility of malignancy when certain features are present, such as a solid component within the TGDC [4]. Given the rarity of TGDC carcinoma, treatment algorithms are not clearly defined [3].

Here, we present a rare case of papillary thyroid cancer present within a thyroglossal duct cyst along with a review of the current literature.

## 2. Case Presentation

A previously healthy 20-year-old female presented to the clinic with a 2-month history of an enlarging submental neck mass. She originally noticed the mass while pregnant. Four weeks after giving birth, however, the area became increasingly more painful and firm to palpation. She began to have pain with swallowing. Physical examination revealed submental and floor of mouth fullness that was exquisitely tender to palpation, limiting the ability to examine her.

**Figure 1:**
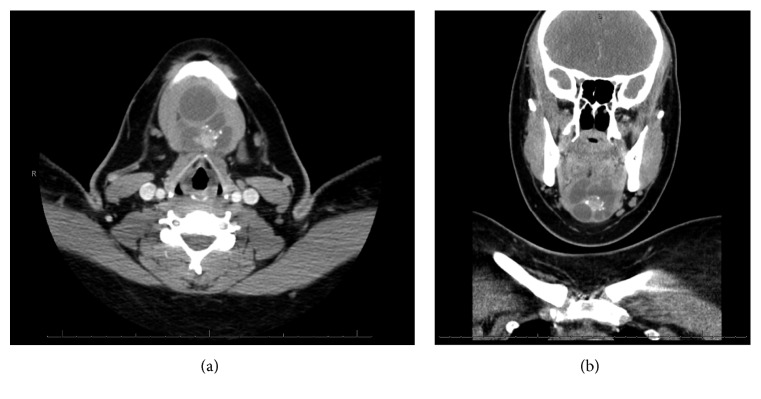
Axial (a) and coronal (b) computed tomography (CT) scan images of submental neck mass.

A neck ultrasound and computed tomography (CT) scan were obtained and showed a multiloculated cystic lesion with calcifications and a central solid component present in the submental space extending down towards the hyoid (Figure 1). There was no radiologic or clinical evidence of thyroid gland disease or neck lymphadenopathy.

A clinical fine-needle aspiration (FNA) was performed to further evaluate the mass and showed mixed inflammatory cells with histiocytes in a background of proteinaceous fluid, consistent with a cyst. Thyroglobulin was significantly elevated within the cystic contents, raising suspicion for malignancy. A repeat FNA with ultrasound guidance was then performed of the solid component of the mass and cytologic preparations demonstrated nuclear overlapping and nuclear grooves with pseudonuclear inclusions, consistent with papillary thyroid carcinoma.

The patient underwent a Sistrunk procedure along with total thyroidectomy. A total thyroidectomy was performed due to the large size of the TGDC carcinoma, the patient's young age, and the likely need for postoperative radioactive iodine therapy. There was no radiographic evidence of lymphadenopathy, and therefore, no neck dissection was planned; however, given the size and location of the mass, contents of level IA and the superior part of level VI were taken with the specimen. A formal level VI neck dissection was not performed due to the negative preoperative imaging.

**Figure 2:**
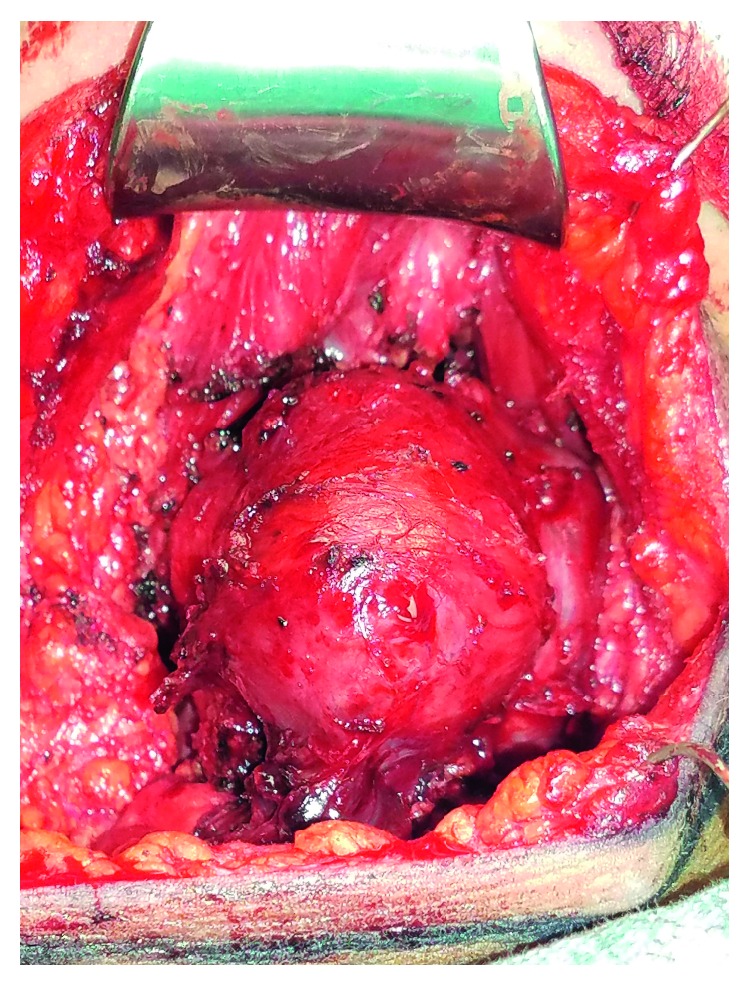
Intraoperative findings of submental neck mass.

Intraoperative findings included a large cystic mass consistent with a TGDC and a small palpable nodule within the thyroid gland (Figure 2). The final surgical pathology was consistent with a pathologic T3aN1a papillary thyroid carcinoma. There was a 6 cm focus of papillary thyroid carcinoma within the TGDC, metastatic papillary thyroid carcinoma in three of six regional nodes with the largest node being 0.2 cm in diameter, and a 0.6 cm focus of papillary thyroid carcinoma within the thyroid gland itself. Her postoperative course was uneventful. She is currently being considered for postoperative radioactive iodine ablation.

## 3. Discussion

The most common presentation of TGDC carcinoma is a rapidly growing and tender neck mass. Malignancy occurs in roughly 1% of TGDCs. Women are more likely than men to have a TGDC carcinoma at a 2.1 : 1 male-to-female ratio. The average age of presentation is in the fourth decade of life [3, 4]. The presence of calcifications within the TGDC and/or regional lymph nodes seen on ultrasound are specific markers for papillary thyroid carcinoma, and the presence of a solid component is suggestive of malignancy [6].

There are two theories as to how a thyroglossal duct cyst carcinoma develops. These include metastasis to the thyroglossal remnant from the thyroid gland versus tumors developing de novo within the remnant. Both seem to be likely, as there are findings supporting both hypotheses. A primary lesion in the thyroid gland is not detected in all cases, which would favor tumor development within the remnant. However, the presence of a primary lesion in the thyroid in one-third or more cases would suggest a metastasis as the cause [5]. This number could be an underestimation, given that not all patients with TGDC carcinoma undergo thyroidectomy [7].

The role of preoperative fine-needle aspiration (FNA) is debated and has an only 53% diagnostic rate for detecting TGDC carcinoma though accuracy of diagnosis is improved by sampling the solid component if present [6]. Overall, TGDC malignancy is relatively uncommon with roughly 278 previously reported cases, the most common malignancy being papillary thyroid carcinoma in 75–80% of cases [4, 8]. Given the rarity, the optimal management is controversial.

It has been well established that the Sistrunk procedure, compared with simple mass excision, has better survival and therefore should be performed in all cases. The role of including total thyroidectomy, however, is not as uniformly agreed upon [6, 9, 10]. It is estimated that thyroid involvement is present in 33–45% of cases of TGDC carcinoma. Some practitioners advocate for total thyroidectomy in all patients with TGDC carcinoma and recommend returning to the operating room to complete this procedure if the carcinoma was not diagnosed until the Sistrunk procedure was completed [10]. Others believe the risks associated with a thyroidectomy procedure are too high to perform in all patients and instead recommend thyroidectomy only in those with higher risk pathologic features or risk factors including known disease spread to lymph nodes, extracapsular invasion, and/or to facilitate postoperative radioactive iodine (RAI) ablation.

It is widely accepted to perform a total thyroidectomy in those patients with known synchronous neoplasms in both the thyroid and TGDC as well as in those patients with high clinical or radiologic suspicion of synchronous tumors [10, 11]. There is also no clear recommendation regarding when to implement RAI treatment. It is appropriate to consider RAI treatment in those patients with large tumors and lymph node involvement or those with malignancy present in both the thyroid and TGDC [10]. The risk of metastatic spread for TGDC carcinoma is low, and therefore elective neck dissection is not generally recommended but should be performed for clinically positive nodal disease [3].

TGDC carcinoma has an excellent survival rate, at 100% and 95.6% at 5 and 10 years, respectively. High-risk characteristics that have been proposed by some include age over 45 years, tumors with a diameter greater than 1.5 cm, previous radiation exposure, presence of nodal disease, invasion of cyst wall, positive histopathologic margins, and presence of a tumor in the thyroid on radiological evaluation [7].

The American Thyroid Association (ATA) guidelines recommend ultrasound monitoring of well-differentiated thyroid carcinoma diagnosed early in pregnancy and to consider surgical intervention if it has grown substantially by 24 weeks or if there is evidence of lymph node involvement. Otherwise, if it remains stable in size or is diagnosed in the second half of pregnancy, then the ATA recommends delaying surgical intervention until after delivery [12]. Our patient had initial presentation of the mass during her pregnancy, but the diagnosis of TGDC carcinoma was not made until after her delivery.

## 4. Conclusion

TGDC carcinoma is a rare entity. As a result, diagnosis can be difficult, as TGDC carcinoma can look identical to benign TGDCs. The extent of treatment is also not well agreed upon. It is widely accepted, however, that a Sistrunk procedure be performed rather than simple excision. The prognosis for TGDC carcinoma is very good. Given the rarity of TGDC carcinoma, case reports and case series are important in order to better understand how best to diagnose and treat TGDC carcinoma.
